# A Journey through the Gonadotropin-Inhibitory Hormone System of Fish

**DOI:** 10.3389/fendo.2017.00285

**Published:** 2017-10-30

**Authors:** José A. Muñoz-Cueto, José A. Paullada-Salmerón, María Aliaga-Guerrero, Mairi E. Cowan, Ishwar S. Parhar, Takayoshi Ubuka

**Affiliations:** ^1^Faculty of Environmental and Marine Sciences, Department of Biology, University of Cádiz, Marine Campus of International Excellence (CEIMAR) and Agrifood Campus of International Excellence (ceiA3), Puerto Real, Spain; ^2^Marine Research Institute (INMAR) – Andalusian Centre of Marine Science and Technology (CACYTMAR), University of Cádiz, Puerto Real, Spain; ^3^Jeffrey Cheah School of Medicine and Health Science, Brain Research Institute, Monash University Malaysia, Bandar Sunway, Malaysia

**Keywords:** LPXRFa, gonadotropin-inhibitory hormone, reproduction, teleosts, GnRH, gonadotropins, gonads, behavior

## Abstract

Gonadotropin-inhibitory hormone (GnIH) is a hypothalamic neuropeptide that belongs to the RFamide peptide family and was first identified in the quail brain. From the discovery of avian GnIH, orthologous GnIH peptides have been reported in a variety of vertebrates, including mammals, amphibians, teleosts and agnathans, but also in protochordates. It has been clearly established that GnIH suppresses reproduction in avian and mammalian species through its inhibitory actions on brain GnRH and pituitary gonadotropins. In addition, GnIH also appears to be involved in the regulation of feeding, growth, stress response, heart function and social behavior. These actions are mediated *via* G protein-coupled GnIH receptors (GnIH-Rs), of which two different subtypes, GPR147 and GPR74, have been described to date. With around 30,000 species, fish represent more than one-half of the total number of recognized living vertebrate species. In addition to this impressive biological diversity, fish are relevant because they include model species with scientific and clinical interest as well as many exploited species with economic importance. In spite of this, the study of GnIH and its physiological effects on reproduction and other physiological processes has only been approached in a few fish species, and results obtained are in some cases conflicting. In this review, we summarize the information available in the literature on GnIH sequences identified in fish, the distribution of GnIH and GnIH-Rs in central and peripheral tissues, the physiological actions of GnIH on the reproductive brain-pituitary-gonadal axis, as well as other reported effects of this neuropeptide, and existing knowledge on the regulatory mechanisms of GnIH in fish.

## Introduction

Gonadotropin-inhibitory hormone (GnIH) is a neuropeptide that was first identified in the Japanese quail (*Coturnix japonica*) brain and exhibited inhibitory actions on gonadotropin secretion both *in vitro* (1) and *in vivo* (2). Following on from pioneer research in avian species, subsequent studies performed in mammals demonstrated that GnIH could also inhibit the reproductive process in this group of vertebrates (3–7). In the last 17 years, GnIH orthologs have been identified not only in tetrapod vertebrates, but also in fish and protochordates (8–10). The ancestral form of GnIH, which has a C-terminal PQRF-amide structure, emerged in the amphioxus, a protochordate species (11). However, this ancestral form of GnIH was duplicated into two paralogous genes, GnIH and NPFF, by chromosome duplication that occurred at the beginning of vertebrate evolution (12, 13). NPFF is also expressed in the brain of vertebrates and discussed when relevant in this review.

Fishes, which represent around half of all living vertebrate species, are one of the most successful radiations in the long evolutionary history of vertebrates (14). Almost all ray-finned fishes are teleosts, which represent the dominant vertebrates inhabiting marine and freshwater ecosystems. Fishes include most commercially important species from fisheries and aquaculture, but also several model organisms for genomics, developmental biology and clinical studies. Despite their impressive biological diversity, key phylogenetic position, economic and scientific importance, the study of GnIH and its physiological effects have been approached in only a few fish species. In addition, the action of GnIH on gonadotropin secretion and reproduction has conflicting results in fish. For example, Amano et al. (15) reported that goldfish GnIH stimulated gonadotropin release from cultured sockeye salmon (*Oncorhynchus nerka*) pituitary cells. On the other hand, Zhang et al. (16) reported that intraperitoneal administration of zebrafish GnIH to goldfish (*Carassius auratus*) inhibited serum gonadotropin levels. The reason for this discrepancy may be partially because the endogenous GnIH peptides of the fish were not used to show their physiological effects. The nature of GnIH effects seems to be dependent on the species, as well as on the sex of the animals, the physiological status, dose, the route and the time elapsed after administration of the GnIH peptide. For example, *in vivo* stimulatory effects of intraperitoneally injected tilapia GnIH (tiGnIH)-2 on FSH and LH secretion have been reported in female tilapia, *Oreochromis niloticus* (17) whereas intracerebroventricular (icv) administration of sea bass GnIH (sbGnIH)-2 inhibited *fshβ* and *lhβ* expression and LH plasma levels in male European sea bass, *Dicentrarchus labrax* (18). In goldfish, there are remarkable differences in reproductive responses to GnIH at different gonadal maturation stages, which reinforce the idea that seasonal reproductive influences are important modulators of GnIH actions (19, 20). Therefore, more efforts on fish GnIH research appear necessary to obtain a clear picture on the role of this neuropeptide on reproduction and other physiological processes in this important group of vertebrates. This review aims at synthesizing the most relevant information regarding the forms, brain distribution, actions and regulation of fish GnIH reported up to date in the literature.

## Comparison of Teleost Fish GnIH with Other Vertebrate GnIH Orthologs

**Figure 1:**
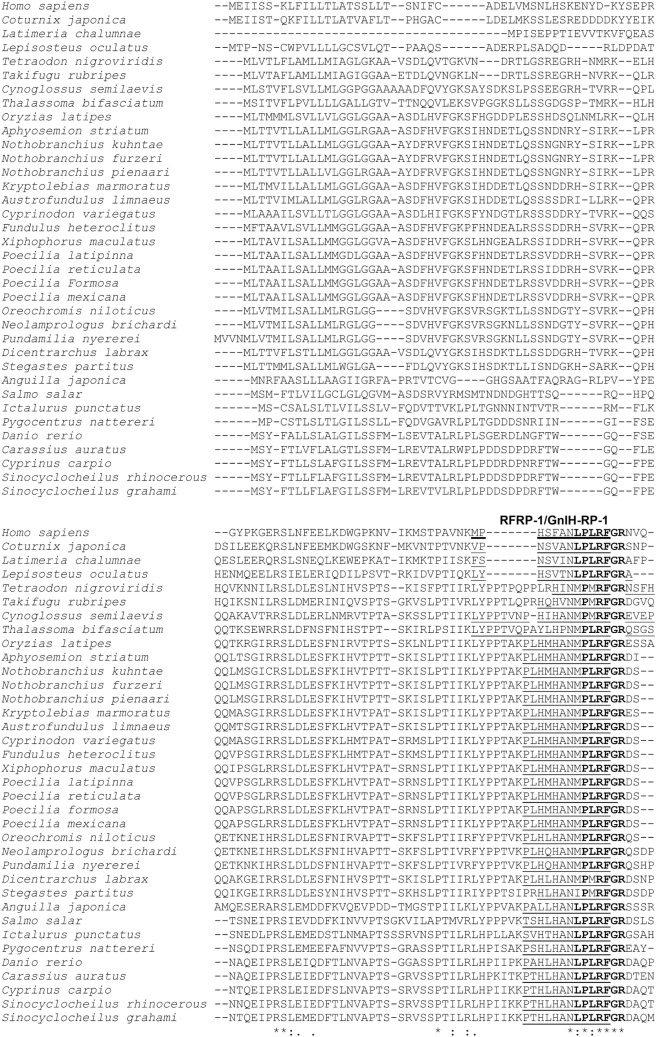

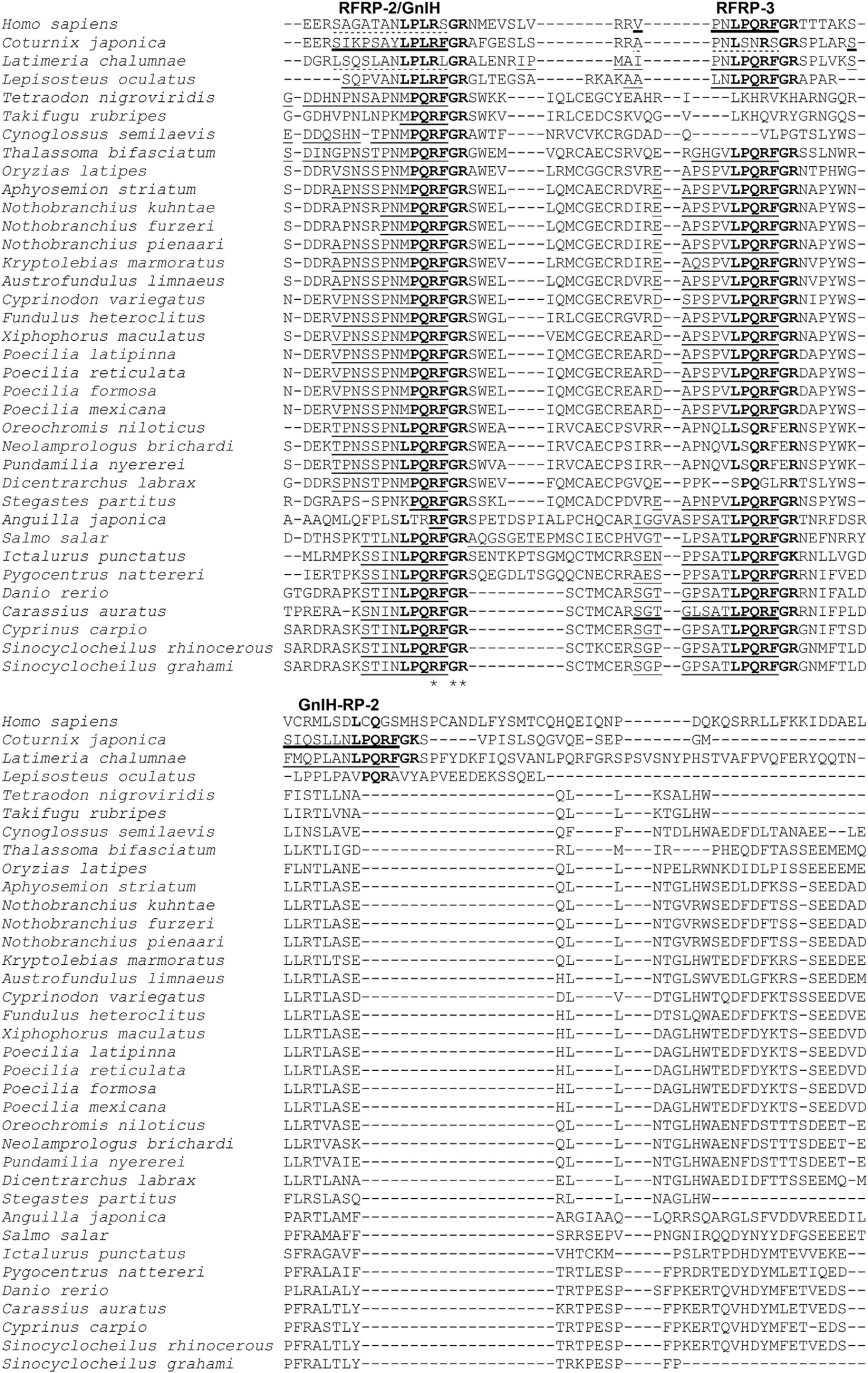

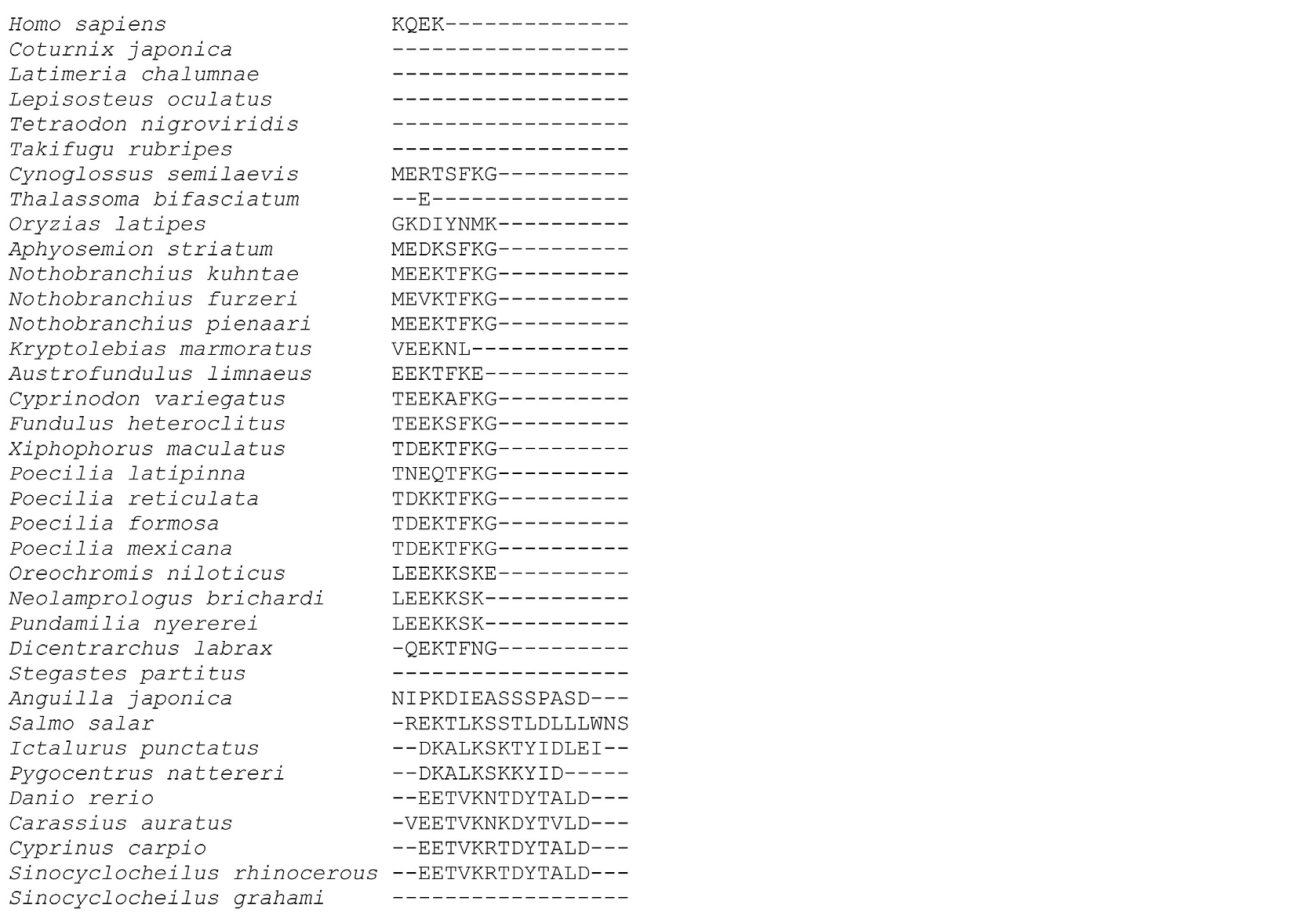
The alignment of gonadotropin-inhibitory hormone (GnIH) precursor polypeptides of human, Japanese quail, coelacanth, spotted gar, and various teleost fish species. The GnIH precursor polypeptide sequences were aligned by using EMBL-EBI Clustal Omega Multiple Sequence Alignment software. The characteristic amino acid sequence of the GnIH peptide, Leu (L), Pro (P), Leu (L) or Gln (Q), Arg (R), Phe (F) with Gly (G) as an amidation signal followed by Arg (R) or Lys (K) as an endoproteolytic basic amino acid at the C-termini are shown in bold. Identified mature GnIH peptide sequences in human (*Homo sapiens*), Japanese quail (*Coturnix japonica*), and goldfish (*Carassius auratus*) are underlined with thick lines. Possible mature GnIH peptide sequences that may be cleaved after the first N-terminal basic amino acids (R or K) are underlined with thin lines. Possible mature GnIH-like peptide sequences that may be cleaved after the first N-terminal basic amino acids (R or K), which have C-terminal—LPLRSamide (human), -LSNRSamide (Japanese quail), and LPLRLamide sequences (*Latimeria chalumnae*, coelacanth) are underlined with broken lines. Accession numbers of GnIH precursor polypeptide sequences in US National Center for Biotechnology Information database are *Homo sapiens* (NP_071433.3), *Coturnix japonica* (XP_015709159.1), *Latimeria chalumnae* (XP_005993154.1), *Lepisosteus oculatus* (XP_015213317.1), *Tetraodon nigroviridis* (BAF34880.1), *Takifugu rubripes* (NP_001092115.1), *Cynoglossus semilaevis* (AMB48604.1), *Thalassoma bifasciatum* (ANV28067.1), *Oryzias latipes* (XP_004073896.1), *Aphyosemion striatum* (SBP35361.1), *Nothobranchius kuhntae* (SBQ91527.1), *Nothobranchius furzeri* (XP_015811406.1), *Nothobranchius pienaari* (SBR89569.1), *Kryptolebias marmoratus* (XP_017278134.1), *Austrofundulus limnaeus* (XP_013866639.1), *Cyprinodon variegatus* (XP_015229614.1), *Fundulus heteroclitus* (XP_012729657.1), *Xiphophorus maculatus* (XP_005802819.1), *Poecilia latipinna* (XP_014884496.1), *Poecilia reticulate* (XP_008419875.1), *Poecilia formosa* (XP_007562706.1), *Poecilia mexicana* (XP_014852162.1), *Oreochromis niloticus* (NP_001298256.1), *Neolamprologus brichardi* (XP_006788138.1), *Pundamilia nyererei* (XP_013765199.1), *Dicentrarchus labrax* (CEK03537.1), *Stegastes partitus* (XP_008290012.1), *Anguilla japonica* (BAV18007.1), *Salmo salar* (XP_013998456.1), *Ictalurus punctatus* (XP_017336524.1), *Pygocentrus nattereri* (XP_017549097.1), *Danio rerio* (NP_001076418.1), *Carassius auratus* (BAC06473.1), *Cyprinus carpio* (AML83913.1), *Sinocyclocheilus rhinocerous* (XP_016370559.1), and *Sinocyclocheilus grahami* (XP_016150344.1). Refer to Table 1 for the common names, class, order, and family of these species. *Indicates positions which have a single, fully conserved amino acid residue, and symbols : and . indicate conservation between groups of strongly (:) and weakly (.) similar amino acid properties, respectively.

The comparison of GnIH precursor and GnIH peptide sequences in various teleost fish species as well as the spotted gar, the coelacanth *Latimeria chalumnae*, the Japanese quail, and humans are presented in Figure 1. In order to identify in this review if the differences in protein or peptide sequences respond to a taxonomic pattern, taxonomic information (Class, Order, Family) of the species analyzed in this figure is provided in Table 1. The mature structure of quail GnIH that was identified by immunoaffinity chromatography and mass spectrometry is SIKPSAYLPLRFamide (1). The cDNA sequence that encodes the quail GnIH precursor polypeptide was cloned and was found to encompass two further peptide sequences besides GnIH that have -LPXRFamide (X = L or Q) sequences at the C-temini. These LPXRFamide peptides were named GnIH-related peptide (RP)-1 and GnIH-RP-2 (21). These three LPXRFamide peptides, GnIH-RP-1, GnIH, GnIH-RP-2 are encoded in the quail GnIH precursor polypeptide in this order (Figure 1). The mature peptide sequence of GnIH-RP-2, which is SSIQSLLNLPQRFamide, was also identified by immunoaffinity chromatography and mass spectrometry (21) (Figure 1). Later in the year 2000, a cDNA encoding human LPXRFamide peptide precursor polypeptide was found in the gene database (22). Human LPXRFamide peptide precursor encompasses one C-terminal—LPLRFamide peptide and one—LPQRFamide peptide in this order, and these were named human RFamide related peptide (RFRP)-1 and -3, respectively. There is also an LPXRFamide-like peptide sequence named human RFRP-2 which has a C-terminal—LPLRSamide sequence in between human RFRP-1 and -3 in the precursor polypeptide (Figure 1). The structures of the mature human RFRP-1 and -3 peptides were identified to be MPHSFANLPLPFamide and VPNLPQRFamide by immunoaffinity chromatography and mass spectrometry (23). The alignment of GnIH precursor polypeptides by the European Bioinformatics Institute (EMBL-EBI) Clustal Omega showed that human RFRP-1 and RFRP-2 align with quail GnIH-RP-1 and GnIH, respectively (Figure 1). Human RFRP-3 aligns with an LPXRFamide-like peptide in the quail GnIH precursor, which has a C-terminal—LSNRSamide sequence (Figure 1).

**Table 1:** Scientific name, common name, class, order, and family of the species analyzed.

Scientific name	Common name	Class	Order	Family
*Homo sapiens*	Human	Mammalia	Primates	Hominidae
*Coturnix japonica*	Japanese quail	Aves	Galliformes	Phasianidae
*Latimeria chalumnae*	Coelacanth	Sarcopterygii	Coelacanthiformes	Coelacanthidae
*Lepisosteus oculatus*	Spotted gar	Actinopterygii	Semionotiformes	Lepisosteidae
*Tetraodon nigroviridis*	Spotted green pufferfish	Actinopterygii	Tetraodontiformes	Tetraodontidae
*Takifugu rubripes*	Torafugu	Actinopterygii	Tetraodontiformes	Tetraodontidae
*Cynoglossus semilaevis*	Tongue sole	Actinopterygii	Pleuronectiformes	Cynoglossidae
*Thalassoma bifasciatum*	Bluehead wrasse	Actinopterygii	Labriformes	Labridae
*Oryzias latipes*	Japanese medaka	Actinopterygii	Beloniformes	Adrianichthyidae
*Aphyosemion striatum*	Red-striped killifish	Actinopterygii	Cyprinodontiformes	Nothobranchiidae
*Nothobranchius kuhntae*	Beira killifish	Actinopterygii	Cyprinodontiformes	Nothobranchiidae
*Nothobranchius furzeri*	Turquoise killifish	Actinopterygii	Cyprinodontiformes	Nothobranchiidae
*Nothobranchius pienaari*	Black rachovii	Actinopterygii	Cyprinodontiformes	Nothobranchiidae
*Kryptolebias marmoratus*	Mangrove rivulus	Actinopterygii	Cyprinodontiformes	Rivulidae
*Austrofundulus limnaeus*	Annual killifish	Actinopterygii	Cyprinodontiformes	Rivulidae
*Cyprinodon variegatus*	Sheepshead minnow	Actinopterygii	Cyprinodontiformes	Cyprinodontidae
*Fundulus heteroclitus*	Mummichog	Actinopterygii	Cyprinodontiformes	Fundulidae
*Xiphophorus maculatus*	Southern platyfish	Actinopterygii	Cyprinodontiformes	Poeciliidae
*Poecilia latipinna*	Sailfin molly	Actinopterygii	Cyprinodontiformes	Poeciliidae
*Poecilia reticulata*	Guppy	Actinopterygii	Cyprinodontiformes	Poeciliidae
*Poecilia formosa*	Amazon molly	Actinopterygii	Cyprinodontiformes	Poeciliidae
*Poecilia mexicana*	Shortfin molly	Actinopterygii	Cyprinodontiformes	Poeciliidae
*Oreochromis niloticus*	Nile tilapia	Actinopterygii	Cichliformes	Cichlidae
*Neolamprologus brichardi*	Princess cichlid	Actinopterygii	Cichliformes	Cichlidae
*Pundamilia nyererei*	Lake Victoria cichlid	Actinopterygii	Cichliformes	Cichlidae
*Dicentrarchus labrax*	European sea bass	Actinopterygii	Perciformes	Moronidae
*Stegastes partitus*	Bicolor damselfish	Actinopterygii	Perciformes	Pomacentridae
*Anguilla japonica*	Japanese eel	Actinopterygii	Anguilliformes	Anguillidae
*Salmo salar*	Atlantic salmon	Actinopterygii	Salmoniformes	Salmonidae
*Ictalurus punctatus*	Channel catfish	Actinopterygii	Siluriformes	Ictaluridae
*Pygocentrus nattereri*	Red-bellied piranha	Actinopterygii	Characiformes	Serrasalmidae
*Danio rerio*	Zebrafish	Actinopterygii	Cypriniformes	Cyprinidae
*Carassius auratus*	Goldfish	Actinopterygii	Cypriniformes	Cyprinidae
*Cyprinus carpio*	Common carp	Actinopterygii	Cypriniformes	Cyprinidae
*Sinocyclocheilus rhinocerous*	horned golden-line barbel	Actinopterygii	Cypriniformes	Cyprinidae
*Sinocyclocheilus grahami*	golden-line barbel	Actinopterygii	Cypriniformes	Cyprinidae

*Scientific name is indicated in the common name section if there is no common name. Classification is based on NCBI Taxonomy Browser besides Dicentrarchus labrax and Stegastes partitus, which are based on World Register of Marine Species*.

The coelacanth is a lobe-finned fish that is closely related to tetrapods. Three LPXRFamide peptides and one LPXRFamide-like peptide are encoded in the coelacanth (*Latimeria chalumnae*) GnIH precursor, and they align to human RFRP-1/quail GnIH-RP-1, human RFRP-2/quail GnIH, human RFRP-3, and quail GnIH-RP-2 (Figure 1). The spotted gar (*Lepisosteus oculatus*) is a ray-finned fish that diverged from teleosts before teleost specific genome duplication, and therefore, it is regarded as a good biomedical model (24). Three LPXRFamide peptides are encoded in the gar GnIH precursor polypeptide and they align to human RFRP-1/quail GnIH-RP-1, human RFRP-2/quail GnIH, and human RFRP-3 (Figure 1). GnIH precursor polypeptides of spotted green pufferfish (*Tetraodon nigroviridis*), torafugu (*Takifugu rubripes*), tongue sole (*Cynoglossus semilaevis*), and European sea bass encode only two LPXRFamide-like peptide sequences, which have C-terminal—MPMRFamide and—MPQRFamide sequences, which align to human RFRP-1/quail GnIH-RP-1 and human RFRP-2/quail GnIH, respectively (Figure 1). GnIH precursor polypeptides of Nile tilapia, princess cichlid (*Neolamprologus brichardi*), and Lake Victoria cichlid *Pundamilia nyererei* encode one LPXRFamide-like and one LPXRFamide peptide sequence, which have C-terminal—MPLRFamide and—LPQRFamide sequences, which align to human RFRP-1/quail GnIH-RP-1 and human RFRP-2/quail GnIH, respectively (Figure 1). The GnIH precursor of the other teleost fish species encodes three LPXRFamide or LPXRFamide-like peptides and they align to human RFRP-1/quail GnIH-RP-1, human RFRP-2/quail GnIH, and human RFRP-3 (Figure 1). The C-terminal sequence of the LPXRFamide-like peptides that aligns to human RFRP-1/quail GnIH-RP-1 is—MPLRFamide in Labriformes, Beloniformes, Cyprinodontiformes, and Cichliformes (Figure 1; Table 1). The C-terminal—LPLRFamide peptides align to human RFRP-1/quail GnIH-RP-1 in Anguilliformes, Salmoniformes, Siluriformes, Characiformes, and Cypriniformes (Figure 1; Table 1). The C-terminal sequence of the LPXRFamide-like peptides that aligns to human RFRP-2/quail GnIH is—MPQRFamide in Tetraodontiformes, Pleuronectiformes, Labriformes, Beloniformes, Cyprinodontiformes, and some Perciformes (Figure 1; Table 1). The C-terminal LPQRFamide peptides align to human RFRP-2/quail GnIH in Cichliformes, Salmoniformes, Siluriformes, Characiformes, and Cypriniformes (Figure 1; Table 1). The C-terminal LPQRFamide peptides align to human RFRP-3 in Labriformes, Beloniformes, Cyprinodontiformes, Anguilliformes, Salmoniformes, Siluriformes, Characiformes, Cypriniformes, and some Perciformes (Figure 1; Table 1).

In summary, most of the teleost GnIH precursor polypeptides encode three LPXRFamide or LPXRFamide-like peptides, which align to human RFRP-1/quail GnIH-RP-1, human RFRP-2/quail GnIH, and human RFRP-3. The C-terminal sequences are mostly conserved in the peptide sequences that align to human RFRP-1/quail GnIH-RP-1, followed by those that align to human RFRP-2/quail GnIH. However, the C-terminal sequence of the peptides that align to human RFRP-3 include all LPQRFamide in teleost fish precursor polypeptides that encode the third LPXRFamide-like or LPXRFamide peptide. The mature teleost fish GnIH peptide structure was only identified in goldfish by immunoaffinity chromatography and mass spectrometry (8), which aligns to human RFRP-3 (Figure 1). The possible N-terminal sequences of teleost fish LPXRFamide or LPXRFamide-like peptides are also well conserved within fish if the peptides were cleaved after the first basic amino acid (Arg or Lys). These results suggest that three or two LPXRFamide or LPXRFamide-like peptides exist in teleost fish. The elucidation of the physiological roles of different endogenous LPXRFamide or LPXRFamide-like peptides should require further investigation in future studies.

## Distribution of GnIH and GnIH Receptors (GnIH-Rs) in Fish

The presence and distribution of GnIH orthologs and their receptors have been explored mainly in the brain but also in peripheral tissues of different vertebrate species by using PCR, immunoassays, immunohistochemistry, and/or *in situ* hybridization. Although the location of GnIH varies among species and the method used for the detection, some features, such as the presence of a periventricular preoptic/hypothalamic GnIH cell population and a profuse brain GnIH innervation, are recurring among studies (25). In mammals, several studies have been performed in rodents, ovine, and primates (including humans), with GnIH cells being identified mainly in the hypothalamus (paraventricular nucleus, dorsomedial nucleus, mediobasal, and ventromedial hypothalamus), as well as in the olfactory bulbs, hippocampus, medulla oblongata, and/or spinal cord, but GnIH is also produced in the eye, testis, and ovary (3, 6, 22, 26, 27). The paraventricular nucleus of the avian and reptile hypothalamus also contains GnIH cells (1, 28–34), in addition the nucleus accumbens and the upper medulla of the Japanese grass lizard was also identified as containing GnIH cells (35). In amphibians, studies carried out in the bullfrog, the European green frog and newt have identified GnIH neurons in the mediobasal telencephalon (medial septum, nucleus of the diagonal band of Broca, and the medial and dorsal pallium) and the diencephalon (anterior preoptic area, suprachiasmatic nucleus, ventral, and dorsal hypothalamic nuclei) (36–39). The pattern of GnIH innervation in the brain of tetrapods is highly consistent with the presence of GnIH-Rs (25, 40). In most tetrapod species studied to date, GnIH neurons project to the median eminence to control anterior pituitary function, and GnIH-Rs are present in the gonadotropes (10, 23, 25, 40). GnIH neurons also project to GnRH-1 and GnRH-2 neurons, which also express GnIH-Rs, at least in birds [GnRH-1 and GnRH-2 neurons; (10, 33, 40)] and hamsters [only GnRH-1 neurons; (7)]. The presence of a profuse GnIH innervation and/or GnIH-Rs outside the hypothalamus and pituitary, from the telencephalon to the rhombencephalon, suggest that GnIH could be involved in functions other than reproduction and could exert pleiotropic actions in vertebrates.

**Figure 2:**
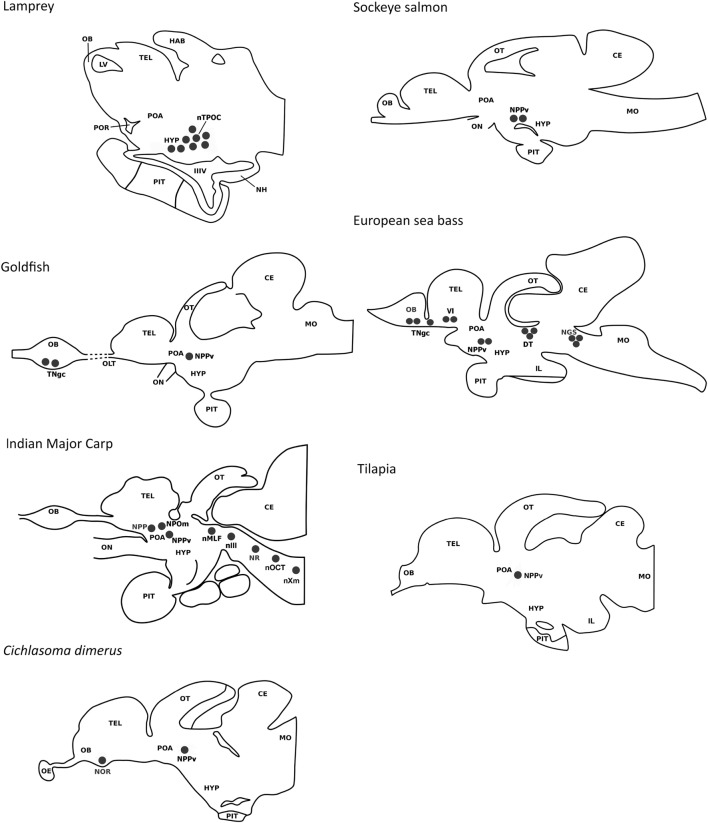
Comparison of the localization of gonadotropin-inhibitory hormone (GnIH) cells in a jawless fish (lamprey) and various teleost species (European sea bass, sockeye salmon, Indian major carp, goldfish, cichlid *Cichlasoma dimerus*, tilapia). GnIH cell populations are represented by black circles. In the lamprey, cells have been reported in the hypothalamic bed nucleus of the tract of the postoptic commissure. In teleosts, the presence of a prominent population of GnIH cells in the posterior periventricular nucleus of the caudal preoptic area is a common feature. GnIH cells have also been reported in the terminal nerve/olfactory bulbs of some teleost species (European sea bass, *Cichlasoma dimerus*, and developing Indian major carp). GnIH cells were also detected in the terminal nerve of the olfactory bulb of goldfish by immunohistochemistry, but not by *in situ* hybridization. Additional GnIH cell masses were detected in the ventral telencephalon of the European sea bass, as well as in the dorsal tegmentum and rhombencephalon of the European sea bass and adult Indian major carp. Abbreviations: CE, cerebellum; DT, dorsal tegmentum; HAB, habenula; HYP, hypothalamus; IIIV, third ventricle; IL, inferior lobe of the hypothalamus; LV, lateral ventricle; MO, medulla oblongata; NGS, secondary gustatory nucleus; NH, neurohypophysis; nIII, oculomotor nucleus; nMLF, nucleus of the median longitudinal fascicle; nOCT, octaval nucleus; NOR, nucleus olfacto-retinalis; NPOm, magnocellular preoptic nucleus; NPP, periventricular preoptic nucleus; NPPv, posterior periventricular nucleus; NR, nucleus reticularis; nTPOC, nucleus of the tract of the postoptic commissure; nXm, motor nucleus of the vagal nerve; OB, olfactory bulb; OE, olfactory epithelium; OLT, olfactory tract; ON, optic nerve; OT, optic tectum; PIT, pituitary; POA, preoptic area; POR, preoptic recess; TEL, telencephalon; TNgc, terminal nerve ganglion cells; Vl, lateral nucleus of the ventral telencephalon.

Despite teleost fishes constituting the most abundant group of vertebrates, studies reporting the distribution of GnIH cells and their projections are scarce and have been addressed in only a few species (Figure 2). In addition, most of these studies have used heterologous antibodies and, only recently, specific antibodies to fish GnIH orthologous peptides have been developed in sea bass and tilapia (41, 42). In goldfish, developing Indian major carp *Labeo rohita*, cichlid fish *Cichlasoma dimerus*, and sea bass, immunohistochemistry has revealed the presence of GnIH-immunoreactive (ir) cells in the terminal nerve/olfactory bulbs (8, 41, 43, 44). However, no labeled cells were detected in the terminal nerve of goldfish by using *in situ* hybridization, which could reflect that GnIH transcripts exhibit low levels or that the antibody used is cross-reacting with another unknown peptide(s) present in this region (8). There are reports showing that NPFF, a sister gene of GnIH, is expressed in the terminal nerve cells of dwarf gourami, *Colisa lalia*, and medaka, *Oryzias latipes* (45, 46). In this sense, the use of a more sensitive technique as laser capture microdissection followed by quantitative real time PCR evidenced that GnIH transcripts can also be detected in terminal nerve GnIH-ir cells of sea bass (41), indicating that immunostained cells in this area represent true GnIH-synthesizing cells, at least in this species. Recently, Corchuelo et al. (47) characterized GnIH in the olfacto-retinal system of zebrafish (*Danio rerio*) by using RT-PCR and qPCR, detecting *gnih* expression in the olfactory epithelium, olfactory bulbs and retina of zebrafish during different stages of oocyte maturation. Moreover, these authors showed inverse expression of *gnih* and *gnrh3* in the olfactory bulbs (47). The presence of GnIH-ir cells in the ventral telencenphalon (Vl) was also reported in the sea bass brain (41). In agreement with these observations, conventional PCR showed important LPXRFamide mRNA levels in the olfactory bulbs/telencephalon of sea bass (41). A conspicuous population of GnIH-ir neurons was consistently detected in the periventricular region of the preoptic area/hypothalamus of all vertebrate groups analyzed, including fish. In the lamprey (*Petromyzon marinus*), the presence of a GnIH cell population has been reported in the hypothalamic bed nucleus of the tract of the postoptic commissure (12). GnIH cells were located in the posterior periventricular nucleus (NPPv) of the caudal preoptic area of goldfish, sockeye salmon, Indian major carp, *Cichlasoma dimerus*, sea bass, and tilapia (8, 15, 41–44), in the ventral zone of the periventricular hypothalamus of zebrafish (48), as well as in the periventricular preoptic nucleus (NPP) and magnocellular preoptic nucleus (NPOm) of Indian major carp (43). Moreover, GnIH-ir perikarya were found in the dorsal mesencephalic tegmentum (close to the nucleus of the medial longitudinal fascicle), as well as in the rhombencephalon of sea bass (secondary gustatory nucleus) and adult Indian major carp (nucleus reticularis, octaval nucleus, and motor nucleus of the vagal nerve) (41, 43). These results are consistent with the important GnIH mRNA levels detected in the midbrain-hindbrain of sea bass by conventional PCR, and laser capture microdissection of caudal GnIH-ir cells followed by qPCR (41), but similar mesencephalic and rhombencephalic cell masses have not been reported in other teleost species.

A common feature between species analyzed is the profuse innervation of GnIH cells within the fish brain, which is particularly evident in the preoptic area, hypothalamus, optic tectum, semicircular torus, and caudal midbrain tegmentum but also has been reported in the olfactory bulbs, ventral/dorsal telencephalon, habenula, pineal, ventral thalamus, vascular sac, pretectum, rostral midbrain tegmentum, posterior tuberculum, reticular formation, and facial-vagal sensory lobe (8, 15, 41–44). The ventral telencephalon, the preoptic area, and the mediobasal hypothalamus of fish have a known presence of GnRH-1 cells, the latter also containing kisspeptin (Kiss2) neurons (49, 50). The presence of GnIH-ir projections and GnIH-Rs have been reported in GnRH-1 cells of mammals (6, 7, 23, 51) and birds (33, 34, 52), as well as on kisspeptin neurons of mammals (53). In zebrafish, LPXRFa fibers interact with GnRH-3 soma from the preoptic area (48) but neither LPXRFa-ir fibers nor LPXRFa-R were found closely associated or coexpressed with GnRH-1, GnRH-3, or Kiss2 neurons in tilapia (42). Whether the association of GnIH with GnRH and kisspeptin neurons exhibits seasonal plasticity in fish or is dependent on the physiological conditions of the animals, as reported in birds (54) should be clarified in future research. But at least in sea bass, diencephalic GnIH expression exhibits marked seasonal variations, with higher transcript levels in the resting season related to the reproductive season (55).

Gonadotropin-inhibitory hormone-ir fibers coursing through the ventral hypothalamus also innervate the fish pituitary, as has been observed in goldfish, sockeye salmon, developing Indian major carp, sea bass, and tilapia (8, 15, 41–43). However, no GnIH-ir fibers or GnIH-ir cells were detected in the pituitary of the cichlid fish *Cichlasoma dimerus* or adult Indian major carp (43). The NPPv of goldfish and sea bass contains hypophysiotropic neurons (41, 56) that could represent the source of GnIH projections reaching the pituitary. In sea bass, DiI tract-tracing and immunohistochemical studies revealed that GnIH cells of the ventral telencephalon could also be the origin of the pituitary GnIH innervation (41). GnIH-ir fibers present in the pituitary of sea bass and tilapia were observed in close proximity of FSH and LH cells of the proximal pars distalis, supporting the neuroendocrine effects of GnIH on gonadotropin synthesis and/or secretion reported in both species (41, 42). Furthermore, most LH cells from the ventral part of the proximal pars distalis were immunolabeled with the tilapia LPXRFa antibody, suggesting that GnIH can also exert autocrine/paracrine effects in the pituitary of this species (42). The sea bass also exhibited GnIH-ir terminals adjacent to GH cells (41), whereas tilapia displayed positive GnIH fibers innervating POMC cells from the rostral pars distalis and α-MSH and somatolactin cells of the pars intermedia (42).

The actions of GnIH are elicited through its binding with GnIH-Rs belonging to the seven transmembrane G protein-coupled receptor family. From the two potential GnIH-Rs identified in vertebrates (GPR147 and GPR74), GPR147 appears to represent the functional receptor for GnIH (25). Less abundant are the studies concerning the identification and distribution of GnIH-Rs in fish. These studies have identified one GnIH-R (GPR147 type) in several species such as the torafugu (31), grass puffer, *Takifugu niphobles* (25), tilapia (17), and sea bass (41), and one GPR147/GnIH receptor was also predicted from gene databases of the coelacanth *L. chalumnae*, spotted gar, Mexican tetra, *Astyanax mexicanus*, rainbow trout, *Oncorhynchus mykiss* and bicolor damselfish, *Stegastes partitus* (25). In tilapia, the binding of tiGnIH to GPR147/GnIH-R activates cAMP/PKA (CRE) and Ca^2+^/PKC (SRE) pathways (17). Interestingly, three different GPR147/GnIH-Rs have been isolated and characterized in two cypriniform species including the zebrafish (16) and the goldfish (57). In zebrafish, receptor activation studies using a heterologous cell-based system, revealed that all three zebrafish LPXRFa peptides activate GPR147/GnIH-R2 and GPR147/GnIH-R3 *via* the PKA/cAMP pathway (48). However, no dose response of the SRE pathway (PKC/Ca^2+^) was detected by any of the three LPXRFa peptides with any of the three GPR147/GnIH-Rs in this species (48). In addition, two different NPFFR receptors from GPR74 subtype were identified in the torafugu, zebrafish and tilapia (16, 17, 31). The effect of medaka GnIH and NPFF on the intracellular cAMP/PKA pathway was investigated in HEK293-T cells transfected with GPR147 or GPR74 subtypes by CRE-luciferase assays (46). GnIH dose-dependently inhibited CRE-luciferase activity stimulated by forskolin in HEK-293-T cells expressing GPR147 at low concentrations from 10^−13^ to 10^−9^ M, but this inhibitory effect diminished dose-dependently at higher concentrations from 10^−9^ to 10^−5^ M. A similar effect of NPFF was observed in HEK293-T cells expressing GPR74-1 or GPR74-2. When GnIH or NPFF was applied without forskolin, a dose-dependent increase in CRE-luciferase activity was observed at concentrations of 10^−8^ M and higher. It is likely that switch of coupling of GPR147/GPR74 from *G*_i_ to *G*_s_ proteins from lower to higher concentrations of GnIH or NPFF may happen in fish (46).

The presence of GnIH-Rs has been identified in central and peripheral tissues of zebrafish, grass puffer, goldfish, and tilapia by RT-PCR (16, 17, 58–60). In zebrafish, the three GnIH receptor genes are expressed in the brain, eye, testis, kidney, spleen, heart, and gill, two of them (*gnih-r1* and *gnih-r3*) are also expressed in the pituitary and one GnIH receptor gene (*gnih-r3*) is present in the ovary (16). In the grass puffer and the tilapia, the GnIH receptor is expressed in the brain and pituitary, the latter species also exhibiting GnIH-R transcripts in the ovary, liver, anterior, and posterior intestine, fat, muscle, and heart (17, 58). GnIH-R mRNA was primarily detected in the brain, pituitary, retina, and gonad of cinnamon clownfish, *Amphiprion melanopus* (60). To date, the precise cellular localization of GnIH-Rs has only been elucidated in the gonads of goldfish by using *in situ* hybridization (59), and in the brain and pituitary of tilapia by using a combination of *in situ* hybridization and immunohistochemistry (17, 42). In goldfish, GnIH-R1 and 2 were localized exclusively to the oocytes before the cortical alveolus stage and to the interstitial tissue to the testis (59). Tilapia GnIH-R-ir cells were distributed widely in the brain, being evident in the olfactory bulbs, dorsal and ventral telencephalic areas, preoptic area, ventral and dorsal thalamus, pretectum, pregromerular area, tuberal hypothalamus, lateral recess, dorsal tegmentum, periventricular gray zone of optic tectum, semicircular torus, posterior tuberal region, granular, and molecular layers of the corpus of the cerebellum, reticular area, superior raphe, and central gray (42). In the tilapia pituitary, immunoreactive GnIH-R cells lie in the dorsal and ventral parts of the rostral pars distalis, and the pars intermedia. In the ventral part of the proximal pars distalis, LH cells were labeled with the GnIH-R antiserum, whereas only a few FSH cells from the dorsal part of the proximal pars distalis appeared immunostained. ACTH cells from the rostral pars distalis and α-MSH cells from the pars intermedia also exhibited GnIH-R immunoreactivity (42). The distribution of GnIH-R-ir cells and/or GnIH-ir fibers in the pituitary of tilapia and sea bass could suggest a role of GnIH not only in reproduction but also in the stress response and growth/metabolism of fish.

## Physiological Actions of GnIH in Fish

**Figure 3:**
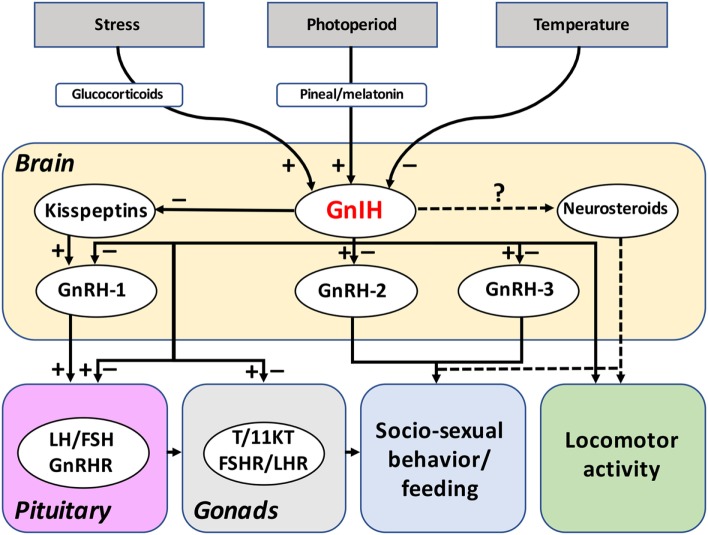
Reported functions and regulation of GnIH system in fish. The reported actions of GnIH on the brain-pituitary-gonad axis of fish are summarized in this figure. Most of the studies have been concentrated on GnIH actions on gonadotropin synthesis and release, evidencing both inhibitory and stimulatory effects. In contrast, only a few studies have addressed the effects of GnIH on neuroendocrine systems and in gonadal physiology in fish. The putative actions of GnIH in fish behavior (socio-sexual behavior, locomotor activity) and feeding could be mediated by its effects on GnRH-2, GnRH-3, kisspeptins, and/or neurosteroids synthesis. Evidences obtained suggest that GnIH could also be mediating the effects of photoperiod, temperature, and stress on the reproductive and other axes of fish, as it has been reported in birds and mammals. Dotted lines refer to suspected actions. Abbreviations: FSH, follicle-stimulating hormone; FSHR, FSH receptor; GnIH, gonadotropin-inhibitory hormone; GnRH, gonadotropin-releasing hormone; GnRHR, GnRH receptor; LH, luteinizing hormone; LHR, LH receptor; T, testosterone; 11KT, 11-ketotestosterone.

The physiological actions of GnIH and related peptides in fish are summarized in Table 2. As it has been described in many studies and reviews, the reproductive inhibitory effects of GnIH were first established in birds and mammals through its reported actions on GnRH and gonadotropin synthesis and secretion (1–3, 61). However, the role of GnIH orthologs in reproduction remains controversial in fish and, as indicated above, the observed inhibitory or stimulatory effects of GnIH could be dependent on the species, sex, reproductive strategies and stages, the peptides used, dose, their route of administration and/or the elapsed time after treatment (Figure 3).

**Table 2:** Molecular structure and physiological actions of GnIH in teleost fish.

Common name of species	Common classification	Putative peptide sequence	Physiological action	Reference
Goldfish	*gfGnIH-1*	PTHLHAN**LPLRFa**	Stimulation of pituitary FSH, LH and GH release (gfGnIH-1,-2,-3, *in vitro*; sockeye salmon)	Amano et al. (15)

			Stimulation of pituitary *lhβ, fshβ, prl*, and *gh* synthesis (gfGnIH-1, *in vitro*; grass puffer)	Shahjahan et al. (58, 62)

			Inhibition of *gnrh-3 and fshβ* synthesis (gfGnIH-2,-3, *in vivo* ip; female goldfish)	Qi et al. (57)

			Inhibition of *lhβ* synthesis (gfGnIH-2, *in vivo* ip; female goldfish)	Qi et al. (57)

			Inhibition of GnRH-stimulated pituitary *lhβ* and *fshβ* synthesis (gfGnIH-3, *in vitro*; mixed sex goldfish)	Qi et al. (57)

	*gfGnIH-2*	AKSNIN**LPQRFa**	Increase of plasma testosterone levels (gfGnIH-2,-3, *in vivo* ip; male goldfish)	Qi et al. (59)

			Stimulation of *star* and *3βhsd* synthesis (gfGnIH-2,-3, *in vivo* ip; male goldfish)	Qi et al. (59)

			Inhibition of *cyp19* synthesis (gfGnIH-2,-3, *in vivo* ip; male goldfish)	Qi et al. (59)

			Stimulation of *fsh-r, lh-r, star*, and *3βhsd* synthesis (gfGnIH-2,-3, *in vitro*; male goldfish)	Qi et al. (59)

			Stimulation or inhibition of LH release, *lhβ, fshβ*, and/or *gnih-r* expression depending on the maturational status and administration route (gfGnIH-3, *in vivo* ip and *in vitro*; mixed sex goldfish)	Moussavi et al. (19, 20, 63)

	*gfGnIH-3*	SGTGLSAT**LPQRFa**	Attenuation of GnRH-2 and GnRH-3 effects on LH secretion and gonadotropin subunit mRNA levels in particular reproductive stages (gfGnIH-3, *in vivo* ip and *in vitro*; mixed sex goldfish)	Moussavi et al. (20)

			Inhibition of GH release and stimulation of *gh* expression (gfGnIH-3, *in vivo* ip; mixed sex goldfish)	Moussavi et al. (63)

			Attenuation of GnRH-2 and GnRH-3 effects on GH release and *gh* expression in a reproductive-dependent manner (gfGnIH-3, *in vivo* ip and *in vitro*; mixed sex goldfish)	Moussavi et al. (63)

			Inhibition of plasma levels of GnRH, FSH and LH, and *sbgnrh, lhβ, fshβ*, and *gthα* expression (gfGnIH-3, *in vivo* ip; immature, male and female cinnamon clownfish).	Choi et al. (60)

			Stimulation of *gnih, gnih-r* and melatonin receptor expression, and of plasma levels of melatonin (gfGnIH-3, *in vivo* ip; immature, male and female cinnamon clownfish)	Choi et al. (60)

Zebrafish	*zfGnIH-3*	SGTGPSAT**LPQRFa**	Decreases in plasma LH level (*in vivo* ip; female goldfish).	Zhang et al. (16)

			Reduction of *gnrh-3* expression (*in vitro*, adult male brain slices of zebrafish).	Spicer et al. (48)

			Downregulation of *lhβ* and common α subunit expression (*in vitro*, adult male pituitary explants of zebrafish)	Spicer et al. (48)

cichlid fish *Cichlasoma dimerus*	*cdGnIH-1*	TPNSSPN**LPQRFa**	Inhibition of LHβ and FSHβ release and stimulation of GH release (cdGnIH-1, *in vitro*; mixed sex cichlid)	Di Yorio et al. (44)

	*cdGnIH-2*	APNQV**LPQRFa**	Stimulation of FSHβ release (cdGnIH-2 *in vitro*; mixed sex cichlid)	Di Yorio et al. (44)

Tilapia	*tiGnIH-2*	QSDERTPNSSPN**LPQRFa**	Stimulation of LH and FSH release (tiGnIH-2, *in vivo* ip and *in vitro*; female tilapia).	Biran et al. (17)

Orange-spotted grouper	*grGnIH-1*	LFPPTAKPFQLHAN**MPMRFa**	Inhibition of *gnrh-1* synthesis (grGnIH-1,-2,-3, *in vivo* ip; female grouper)	Wang et al. (64)

	*grGnIH-2*	ESVPGDDSAPNSTPN**MPQRFa**	Inhibition of *lhβ* synthesis (grGnIH-2, *in vivo* ip; female grouper)	Wang et al. (64)

	*grGnIH-3*	EAQNPI**LPQRL**	Stimulation of *gnrh-3* synthesis (grGnIH-3, *in vivo* ip; female grouper)	Wang et al. (64)

			Stimulation of *lh-r* synthesis (grGnIH-2, *in vitro*; female grouper)	Wang et al. (65)

			Stimulation of *star* and *3*β*hsd1* synthesis (grGnIH-1, *in vivo* ip and *in vitro*; female grouper)	Wang et al. (65)

Sea bass	*sbGnIH-1*	PLHLHAN**MPMRFa**	Inhibition of brain *gnrh2, kiss1, kiss2, kiss1-r, gnih*, and *gnih-r* expression (sbGnIH-2 *in vivo* icv; male sea bass)	Paullada-Salmerón et al. (18)

	*sbGnIH-2*	SPNSTPN**MPQRFa**	Inhibition of brain *gnrh-1* expression (sbGnIH-1 *in vivo* icv; male sea bass)	Paullada-Salmerón et al. (18)

			Inhibition of pituitary *fshβ, lhβ, gh*, and *gnrh-r-II-1a* expression (sbGnIH-1,-2 *in vivo* icv, im; male sea bass)	Paullada-Salmerón et al. (18, 66)

			Decreases in plasma LH levels (sbGnIH-1,-2 *in vivo* icv, im; male sea bass)	Paullada-Salmerón et al. (18, 66)

			Decreases in plasma FSH levels (sbGnIH-1 *in vivo* im; male sea bass)	Paullada-Salmerón et al. (66)

			Decreases in plasma T and 11-KT levels (sbGnIH-1, 2 *in vivo* im; male sea bass)	Paullada-Salmerón et al. (66)

			Increases in diurnal activity (sbGnIH-1,-2 *in vivo* im; male sea bass)	Paullada-Salmerón et al. (66)

*Characteristic C-terminal five amino acid sequences are highlighted in bold. icv, intracerebroventricular injection; im, intramuscular injection; ip, intraperitoneal injection*.

### GnIH Actions on Neuroendocrine Systems in Fish

The presence of profuse GnIH fiber projections and/or their receptors in the ventral telencephalon, the preoptic area, and the hypothalamus of several fish species (8, 15, 41–43, 57) suggests that GnIH might be acting at the central level to modulate the main neuroendocrine systems controlling the reproductive process. To date, most of the reported effects of different GnIH orthologs on brain neuroendocrine systems are inhibitory (Table 2). For example, icv injection of sbGnIH-1 in mature male sea bass, and intraperitoneal (ip) injections of grouper GnIH (grGnIH)-1, -2, and -3 in female grouper (*Epinephelus coioides*), and of goldfish GnIH (gfGnIH)-3 in inmature, male and female cinnamon clownfish decreased *gnrh-1* mRNA levels, the latter species also exhibiting reduced plasma GnRH levels after GnIH treatment (41, 60, 64). In mature male sea bass, the effects of GnIH on GnRH-2 expression appear dependent of the route of administration because centrally (icv)-administered sbGnIH-2 reduced *gnrh-2* mRNA levels, whereas the same GnIH form injected peripherally (intramuscular) increased the *gnrh-2* expression (18, 66). In turn, gfGnIH-2 and gfGnIH-3 peptides inhibited the expression of *gnrh-3* gene, having no effects on *gnrh-2* transcript levels in female goldfish (57), and zebrafish GnIH (zfGnIH)-3 reduced *gnrh-3* expression in brain slices of zebrafish (48), but stimulatory effects of grGnIH-3 on *gnrh-3* expression have been reported in ip-injected female groupers (64). The kisspeptin system of sea bass is also modulated by GnIH where sbGnIH-2 icv-injected animals exhibited decreased *kiss1, kiss2*, and *kiss1r* transcript levels (18). These results are consistent with the presence of abundant GnIH-ir fibers in the habenula, preoptic area, rostral mediobasal hypothalamus and around the lateral recess of sea bass, where Kiss1 and Kiss2 neurons are present (41, 50). Results obtained in cinnamon clownfish and sea bass showed that GnIH can also modulate brain *gnih* and *gnih-r* mRNA levels, indicating the existence of an autocrine regulation on the brain GnIH system (18, 41, 60). However, the nature of these autoregulatory actions seems to be dependent on the route of administration because centrally (icv)-injected sbGnIH-2 decreased *gnih* and *gnih-r* expression in sea bass (18), whereas peripherally (intramuscular or intraperitoneal)-administrated sbGnIH-2 and gfGnIH-3 increased *gnih* and *gnih-r* mRNA levels in cinnamon clownfish and sea bass, respectively (41, 60).

### Role of GnIH in Fish Pituitary

The distribution pattern of GnIH fibers and its receptors described above reinforces the involvement of this neuropeptide in the regulation of pituitary hormone synthesis and secretion in fish (Figure 3). The first physiological study developed in teleost fish demonstrated that *in vitro* treatment of cultured pituitary cells with exogenous GnIH (goldfish GnIH-1, -2, and -3 peptides) stimulated the release of LH and FSH in sockeye salmon (15). Likewise, *in vitro* treatment with gfGnIH-1 enhanced *fshβ* and *lhβ* gene expression in grass puffer pituitary (58) and *Cichlasoma dimerus* GnIH (cdGnIH)-2 peptide also provoked an increase of *in vitro* FSH-β release in intact pituitary cultures of this cichlid fish (44). In female tilapia, both *in vivo* and *in vitro* studies revealed that tiGnIH-2 induced a significant increase in FSH and LH secretion (17).

On the other hand, *in vivo* and *in vitro* studies performed in goldfish examining the effects of gfGnIH-3 on pituitary *lhβ, fshβ*, and *gnih-r* mRNA levels, as well as on LH secretion showed mixed results (19, 20). Intraperitoneal (ip) injection of gfGnIH-3 peptide reduced LH plasma levels at early- and mid-gonadal recrudescence, as well as the expression of *gnih-r* mRNA levels in the pituitary at mid-late and late recrudescence stages, but increased the mRNA levels of both *lhβ* and *fshβ* at early to late recrudescence. Furthermore, the incubation of cultured pituitary cells with gfGnIH-3 provoked a decrease of *lhβ* and *fshβ* transcript levels at early and late recrudescence, but elevated the gene expression of *lhβ* at mid-recrudescence and stimulated the LH secretion at late-gonadal recrudescence. Moussavi and colleagues have further investigated the interaction of gfGnIH-3 with two forms of GnRH, GnRH-2 and GnRH-3, both *in vivo* and *in vitro* (20). Administration of gfGnIH-3 or GnRH alone evoked an increase of *lhβ* and *fshβ* mRNA levels at early-, mid-, and late-gonadal recrudescence, but gfGnIH-3 treatment showed no effect on transcription of *lhβ* gene at late recrudescence. Conversely, co-injection of gfGnIH-3 with GnRH-3 reduced the expression of *lhβ* mRNA levels at early recrudescence, and *lhβ* and *fshβ* mRNA levels during mid- and late-gonadal recrudescence, whereas the co-treatment of gfGnIH-3 with GnRH-2 decreased the transcript levels of *lhβ* at mid and late recrudescence. Additionally, during early- and mid-gonadal recrudescence, treatment with gfGnIH-3 caused an inhibition of plasma LH levels, and this reduction was also observed in fish co-injected with GnRH-2 or GnRH-3 in early gonadal recrudescence. Treatments with GnRH-2 and GnRH-3 stimulated *gnih-r* expression at mid and late recrudescence, while opposite effects, i.e., reduced *gnih-r* mRNA levels, were observed after gfGnIH-3 treatment during late recrudescence. *In vitro* experiments showed that exposure to gfGnIH-3 suppressed GnRH-2-induced LH response at mid recrudescence. Taken together, these results suggest that GnIH in goldfish can exert complex stimulatory or inhibitory effects on gonadotropin synthesis and secretion, as well as on GnRH regulation of this pituitary function, depending on the reproductive stage of the animals and administration route (19, 20).

In contrast, inhibitory effects of GnIH on gonadotropins have been reported in other studies performed in species such as goldfish, cinnamon clownfish, cichlid *C. dimerus*, orange-spotted grouper, sea bass, and zebrafish (16, 18, 44, 48, 57, 60, 64, 66). The ip administration of zfGnIH-3 decreased LH plasma levels in female goldfish at 1 and 3 h post injection at the highest doses tested (16). Another *in vivo* study performed in female goldfish showed that animals treated with gfGnIH-2 and gfGnIH-3 peptides had reduced *fshβ* mRNA levels in the pituitary gland, while only the gfGnIH-2 form was able to decrease *lhβ* expression significantly (57). Nevertheless, *in vitro* administration of gfGnIH-2 and gfGnIH-3 in cultured pituitary cells, although it had no effect on either *fshβ* or *lhβ* mRNA levels, decreased GnRH-stimulated *fshβ* and *lhβ* expression (57). In the pituitary gland of the female grouper, injections of grGnIH-2 peptide decreased *lh*β mRNA levels (64). In 2016, Choi and collaborators also evaluated the effects of GnIH on transcript and plasma levels of gonadotropins in immature, and mature male and female cinnamon clownfish showing that ip injection with heterologous gfGnIH-3 reduced *gthα, fshβ*, and *lhβ* expression, as well as plasma FSH and LH (60). In the cichlid fish *Cichlasoma dimerus*, cdGnIH-1 peptide also inhibited LHβ and FSHβ release in intact pituitary cultures (44). Recent studies performed in the male sea bass showed that treatment with both sbGnIH-1 and sbGnIH-2 peptides exerted inhibitory actions on the synthesis and release of gonadotropins (18, 66). Findings revealed that the central (18) and peripheral (66) administration of sbGnIH-1 or sbGnIH-2 peptides reduced LH plasma levels, as well as pituitary *lhβ* mRNA levels. Furthermore, only the icv injection with sbGnIH-2 determined a reduction of pituitary *fshβ* and *gnrh-r-II-1a* receptor mRNA levels (18), whereas intramuscular administration of sbGnIH-1 elicited a decrease in FSH plasma levels (66). In pituitary explants, zfGnIH-3 downregulated *lhβ* and common α subunit expression (48).

In addition to its effects on gonadotropins, GnIH has also been shown to be involved in the regulation of the synthesis and/or release of other adenohypophyseal hormones in fish. A pioneer study performed in sockeye salmon showed that *in vitro* treatment of cultured pituitary cells with the three goldfish GnIH peptides (gfGnIH-1, -2, and -3) stimulated GH release (15). Accordingly, *in vitro* gfGnIH-1 increased *gh* transcript levels, as well as those of prolactin, in grass puffer (62) and cdGnIH-1 augmented the release of GH in pituitary cultures of a cichlid species (44). However, ip injection of tiGnIH-2 did not show any change in GH release in female tilapia (17). Another study in goldfish showed that ip injection of gfGnIH-3 reduced GH plasma levels at early, mid, and late recrudescence, but elevated *gh* gene expression in the pituitary (63). These authors also showed that gfGnIH-3 attenuates the effects of GnRH-2 and GnRH-3 on GH release and *gh* expression in a reproductive-related manner (63). Icv injections of sbGnIH-2 inhibited *gh* mRNA levels in a dose dependent manner in male sea bass (18). The presence of GnIH fibers in the proximal pars distalis of the sea bass pituitary, innervating GH-ir cells, and in the pars intermedia (41), as well as coursing close to somatolactin, α-MSH and ACTH cells of tilapia, the two latter cell types also exhibiting GnIH-receptor immunoreactivity (42), reinforces the assumption that GnIH can modulate the synthesis and secretion of some pituitary hormones other than gonadotropins.

### Role of GnIH on Gonadal Physiology

It has been demonstrated that not only the brain, but also the gonads may be a source of GnIH. Based on different studies in birds and mammals (67–71), GnIH has also been considered as a peripheral regulator of gonadal functions. The fish gonads also synthesize GnIH, as reflected in different studies showing *gnih* expression in the ovary and/or the testis of zebrafish, tilapia and sea bass (16, 17, 41, 47). Recently, *gnih* transcripts were identified in the cortical vesicles of previtellogenic oocytes of zebrafish, as well as in the follicular cells and in the zona radiata of the vitellogenic oocytes (47). Although the expression of GnIH and GnIH-R has been reported in the gonads of different teleost species (16, 17, 41, 47, 59, 65), most of the research has been focused on GnIH actions in gonadotropin synthesis and secretion, and only a few studies have addressed the role of GnIH in gonadal gametogenesis and/or steroidogenesis of fish (59, 65, 66). Qi and collaborators (59) studied the effects of GnIH on steroidogenesis in male and female goldfish gonads. Implantation of gfGnIH-2 or gfGnIH-3 did not induce any change in estradiol plasma levels in females, but both GnIH peptides increased serum testosterone levels in male goldfish. Additionally, ip injection with both GnIH forms enhanced the gene expression of *steroidogenic acute regulatory protein* (*star*) and *3β-hydroxysteroid dehydrogenase* (*3βhsd*) genes and decreased *cyp19* mRNA levels in the testes of the goldfish. *In vitro* analysis using gfGnIH-2 and gfGnIH-3 in cultured testicular cells also significantly increased *fshr, lhr, star*, and *3βhsd* transcript levels, but reduced *cyp19* expression. However, ovarian mRNA levels of gonadotropin receptors and steroid-synthesizing enzymes were unaffected after both *in vivo* and *in vitro* GnIH treatments (59). In contrast, in a recent study performed in female grouper, treatment with grGnIH-2 increased *lhr* mRNA levels in cultured ovary fragments, and grGnIH-1 peptide stimulated the ovarian expression of *star* and *3βhsd* both *in vitro* and *in vivo* (65). On the other hand, the peripheral sbGnIH-1 and sbGnIH-2 implants in male European sea bass caused an inhibition of testosterone and 11-ketotestosterone plasma levels at particular reproductive stages (early- and mid-spermatogenesis), without affecting plasma levels of the progestin 17,20β-dihydroxy-4-pregnen-3-one (66). Moreover, intramuscular implant of both GnIH peptides determined a delay in the development of testis, which exhibited abundant type A spermatogonia (SgA) and only scattered and isolated clusters of spermatozoids at the spermiation phase (66). Taken together, these findings suggest that GnIH may regulate the reproductive axis of teleost fish by acting not only at the brain and pituitary levels but also on gonadal physiology, the nature of its actions depending on the species, the sex and the reproductive stage (Figure 3).

### Role of GnIH in Fish Behavior

First reports of GnIH effects on socio-sexual behavior and/or sexual motivation were obtained in birds (34, 72, 73) and mammals (51, 74). Despite many studies that have explored the interactions between GnIH and the reproductive axis, how GnIH is involved in the regulation of reproductive, social and other behaviors in fish is still almost unknown. To date, the only study addressing the role of GnIH in fish behavior has been accomplished in the European sea bass (66), showing that both sbGnIH-1 and sbGnIH-2 affected the diurnal to nocturnal ratio of locomotor activity along the reproductive cycle. In this study, whereas control animals progressively decreased their diurnal habits as spermatogenesis progressed and they reached the spermiation phase, GnIH treatment induced a significant elevation in their diurnal pattern of activity along the same gametogenic stages, and this diurnal activity only decreased when the GnIH treatment ceased at the spawning season (66). It is interesting to note that the European sea bass is a rhythmic species that exhibits diurnal feeding and locomotor activity patterns during most of the year, but switches to nocturnal during the reproductive season at winter (75, 76). Moreover, diencephalic expression of *gnih* in sea bass was higher during the resting season and lower during the reproductive season (55). Therefore, it is plausible to consider that this seasonal pattern of GnIH expression could be on the basis of this natural diurnal to nocturnal shift observed in the reproductive season of this species.

As indicated above, the midbrain of Indian major carp and European sea bass contains a GnIH cell population located in the dorsal mesencephalic tegmentum (41, 43). Based on their location and the profuse GnIH innervation in sensory-motor areas, a role in regulating behavior (locomotor activity, mating, feeding) may be considered for this GnIH cell population in sea bass (41, 77). GnIH has been found to be involved in the modulation of socio-sexual behavior in birds by acting on tegmental GnRH-2 cells, and promoting the conversion of testosterone into neuroestrogens *via* the stimulation of brain cytochrome P450 aromatase activity (10, 34, 73). Furthermore, kisspeptin and GnIH seem also to interact in the regulation of social behavior in mammals and teleosts (78). Interestingly, both sbGnIH-1 and sbGnIH-2 forms decrease plasma testosterone levels, and sbGnIH-2 modulates brain *gnrh2* and *kisspeptin* gene expression in sea bass (18, 66). Several studies performed in fish have reported the potential involvement of neurosteroids in socio-sexual behaviors. For instance, in *Lythrypnus dalli*, a socially induced decrease in brain aromatase levels resulted in increased aggression (79). In turn, treatments with aromatase inhibitors decreased aggressive behavior in African male cichlid fish *Astatotilapia burtoni* (80) and reduced courtship activities in male Endler guppy *Poecilia reticulata* (81). Altogether, these results suggest that GnIH might also be involved in the regulation of reproductive, social and/or locomotor behaviors in fish through its actions on GnRH-2, kisspeptins and/or neurosteroid synthesis and release (Figure 3).

## Regulation of the GnIH System in Fish

A range of evidence indicates that the GnIH system is mediating the effects of photoperiod on different physiological processes in Tetrapods, with results in photoperiodic mammals, birds and amphibians suggesting that its expression is modulated through a melatonin-dependent process (7, 32, 38, 82, 83). Unfortunately, much less is known regarding the regulatory mechanisms of GnIH in fish, although scarce data available support the idea that environmental cues such as photoperiod and temperature are also regulating daily and seasonal profiles of GnIH in this group of vertebrates (Figure 3).

In general, photoperiodic regulation of reproduction in fish is mediated by plasma melatonin release from the pineal gland, acting at all levels of the reproductive axis (84, 85). Data collected in fish also suggest that melatonin might exert its action, at least in part, through GnIH neurons (Figure 3). A recently published study analyzed the relationship between melatonin and GnIH in the cinnamon clownfish, and reported that GnIH was co-localized with the melatonin receptor MT-R1 in diencephalic cells (60). Likewise, GnIH cells in sea bass are located in regions known to exhibit melatonin-binding sites (41, 86). Bidirectional connections between the pineal organ and GnIH cells appear to exist in fish, because the pineal organ projects to the NPPv and the dorsal tegmental area, where GnIH cells have been identified in teleosts (87–89), and GnIH-ir fibers have been identified in the fish pineal organ (41). In addition, in grass puffer, the expression of *gnih* and its receptor showed diurnal and circadian rhythmicity at the spawning stage, in association with melatonin receptor expression, suggesting that the action of GnIH is cyclic possibly due to regulation by melatonin and the functional role of the GnIH system is in the regulation of lunar-synchronized spawning (58, 90). Interestingly, Cowan and co-workers reported, for the first time in fish, that GnIH gene expression is regulated by the pineal organ. Their findings in sea bass revealed that pinealectomy (Px) reduced the expression of *gnih* in a regional- (in mid-hindbrain, but not in the telencephalon or diencephalon) and reproductive- (in reproductive season but not in resting) dependent manner (55). Moreover, a seasonal difference in *gnih* and *gnih-r* expression was observed in the diencephalon, where both Px and control groups exhibited higher transcript levels of these genes at resting than in the reproductive season (55). All these evidences support the hypothesis that melatonin could play an important role in the regulation of the GnIH system in fish, which could mediate, in turn, in the transduction of environmental information to other reproductive-related systems.

In fish, increasing evidence supports the decisive role of photoperiod and/or temperature on larval development, sex determination/differentiation and puberty (91–94). However, ontogenetic studies of the GnIH system and its developmental regulation by environmental factors have not been adequately addressed in this vertebrate group. In a recent study, Paullada-Salmerón and colleagues performed the first analysis of *gnih* and *gnih* receptor expression pattern throughout the first year of life in the European sea bass (95). This study revealed that both *gnih* and *gnih-r* showed significantly increased expression from hatching to the second/third week of life, a subsequent decrease in mRNA levels until 120 days post-fertilization (dpf) and then a further increase at the onset of sex differentiation (150 dpf). Afterward, *gnih* transcript levels dropped significantly during the sex differentiation period (150–240 dpf) and continued at that level for the remainder of that year. The results also revealed daily variations in developmental expression of *gnih* and *gnih-r*, with higher diurnal mRNA levels at early stages (until 25 dpf), and a shift to higher nocturnal expression at 300–360 dpf coinciding with the winter (reproductive season) (95). In the same study, Paullada-Salmerón and co-workers further investigated the effects of rearing temperature during the thermosensitive period on the expression of the *gnih* and its receptor in sea bass. Early exposure to high temperatures (21°C), which is known to provoke a masculinization of the progeny (96), decreased the levels of *gnih* and *gnih-r* transcripts during early development and these effects were also evident at the end of the sex differentiation period (240–300 dpf), indicating that temperature can exert remarkable effects on the transcription of both genes (95). Altogether, these results could indicate that the GnIH system might not only be involved in the modulation of the reproductive cycle but could also be a mediator in sex differentiation and puberty in fish. Involvement of GnIH in the regulation of reproductive development and puberty has been also studied in birds and mammals (97, 98).

Several findings suggest that stress may act through GnIH neurons to inhibit reproductive function in birds and mammals (10, 99). Both acute and chronic stress upregulate hypothalamic GnIH gene expression in rats, and this stress-induced increase of GnIH is blocked by adrenalectomy (100). Glucocorticoid receptors are present in GnIH neurons, as revealed by immunohistochemistry, and could sustain these effects (101). In quail and mice, corticosterone induces GnIH expression *via* the glucocorticoid receptor present in GnIH neurons and these actions appear to be mediated by the glucocorticoid-response element (GRE) present in the promoter of the GnIH gene (101). Although there is no report indicating the involvement of GnIH in stress response in fish, evidence suggests that a similar mechanism to that reported in Tetrapods may be operating in fish (Figure 3). Promoter prediction searching has revealed the presence of several putative GRE in the zebrafish *gnih* and *gnih-r* gene promoter sequences (25). Stimulatory effects of cortisol on *gnih* transcript levels have been reported in the cinnamon clownfish (102). The presence of GnIH-ir fibers in close proximity to α-MSH and ACTH cells, which also exhibit GnIH-R immunoreactivity, was identified recently in the pituitary of tilapia (42). These results indicate that the role of GnIH in the mediation of the stress response could be evolutionarily conserved in vertebrates.

## Concluding Remarks

Increasing research is showing that GnIH is present throughout the vertebrate lineage from fish to mammals. Most of the teleost GnIH precursor polypeptides identified to date encode three LPXRFamide or LPXRFamide-like peptides, although some species exhibit only two LPXRFamide-like peptide sequences. The presence of a diencephalic GnIH cell population in the preoptic area/hypothalamus, the profuse GnIH innervation in the brain and the hypophysiotropic character of GnIH seem also to represent a common feature for all vertebrate groups, including fish. However, recent studies in teleosts are revealing that GnIH cells can also be found in cells from the olfactory bulbs, ventral telencephalon, dorsal midbrain tegmentum, and rostral rhombencephalon, likely to be coexisting with other RFamide neuropeptides. Functional studies of GnIH in fish have mainly focused on its effects on gonadotropin synthesis and secretion, but increasing evidence is showing that it can also regulate reproduction by modulating brain GnRH and kisspeptin systems, as well as gonadal gametogenesis and steroidogenesis. Contrary to that referenced in birds and mammals, in which inhibitory actions of GnIH represent the main feature, both inhibitory and stimulatory actions of GnIH have been reported in the reproductive axis of fish (Figure 3). This diversity in actions could be related to the species, the sex of the animals, the physiological status, the route, and the time elapsed after administration of the GnIH peptide. Nevertheless, the results obtained in a recent dose-response study (18), as well as in work analyzing the ligand dose-dependent activation of GPR147/GPR74 receptors by GnIH and NPFF (46), suggest that the GnIH actions on the reproductive axis of fish could be inhibitory at low (physiological) concentrations, and stimulatory at higher (pharmacological) concentrations, which could also explain the diversity of GnIH actions reported in fish studies. Whether centrally- and peripherally- (e.g., gonadal) synthesized GnIH exert different effects on reproduction and other physiological processes remains an open question. Reinforcing and building on the knowledge acquired in the last decade on GnIH in fish will require further efforts to clarify the role of this RFamide neuropeptide in functions other than reproduction, such as feeding, stress response, and behavior, as well as in elucidating the intracellular pathways and regulatory mechanisms involved in GnIH actions.

## Author Contributions

All authors listed have made a substantial, direct, and intellectual contribution to the work and approved it for publication.

## Conflict of Interest Statement

The authors declare that the research was conducted in the absence of any commercial or financial relationships that could be construed as a potential conflict of interest.
